# Visualizing Complex Problems in Leadership Education: A System Dynamics Perspective

**DOI:** 10.1002/yd.70019

**Published:** 2025-12-07

**Authors:** Catlin M. Goodwin, Tiffany A. Marzolino

**Affiliations:** ^1^ Department of Community Sustainability Michigan State University Michigan USA; ^2^ Agricultural Leadership, Education & Communications Program University of Illinois Urbana‐Champaign Illinois USA

## Abstract

As the challenges addressed in leadership education become increasingly complex, it is essential to develop and employ new skills and tools which help individuals and groups make sense of the complexity. In this paper, we introduce system dynamics as a lens for leadership education stakeholders to understand and navigate complex problems and systems. Then, we demonstrate two practical entry points into system dynamics practice. Through thick description and an example case study, we conclude by describing how to use and facilitate rich pictures and causal loop diagrams to make sense of complex problems.

## 1 Introduction

We begin with a fictional story about an educator named Rachel. She loves her job, but it has its challenges, one of which stems from an idea to incorporate a community‐engagement project in one of her courses. Rachel knew the project was going to encounter significant hurdles. The university had weak relationships with community organizations, and the students, inexperienced with community‐engaged work, would have many questions she would have to respond to, without much precedent. As expected, the first run of the project was challenging, but she learned a lot, made some changes, and tried again. What Rachel did not expect was three iterations of the project later, she would still face significant challenges finding community partners with suitable projects, ensuring mutual benefit for community partners and students, and, most challenging, motivating students to take initiative and engage fully.

We've all been there—we see an opportunity with great potential, but no matter what strategies we implement, we run into persistent challenges. Literature and experience tell us persistent problems are signs of complexity (Meadows 2008) and, try as we might, we cannot fix complex problems with simple solutions (Marion and Uhl‐Bien 2001). We need another way of thinking to help us see the problem for what it is: the product of a series of interactions across a complex system. Numerous scholars across the leadership education landscape have called for systems‐oriented thinking in practice and research (Boylan and Turner 2017; Cletzer and Kaufman 2020; Satterwhite et al. 2020; Watkins et al. 2017). Developing complex problem‐solving skills is described as necessary for progressing leadership education and navigating our increasingly complex, global environments (Satterwhite et al. 2020).

The purpose of this article is to introduce system dynamics as a lens for students, faculty, and other stakeholders to explore complex problems in leadership education. First, we situate the core elements of system dynamics in the leadership education context, creating a shared understanding of common system components. Then, we describe two practical tools; rich pictures (Bell et al. 2016) and causal loop diagrams (CLDs) (Barbrook‐Johnson and Penn 2022), for visualizing complex systems. Finally, we share insights and strategies to support meaningful change with system dynamics tools. Throughout the paper, we use the story of our fictional colleague, Rachel, to illustrate the use of system dynamics. However, we also encourage you, the reader, to consider the persistent challenges you interact with and how the system dynamics lens might help you intervene.

## 2 Situating System Dynamics

System dynamics is a systems‐thinking method used to develop heuristic tools which support problem solving and understanding system behavior (Ford 2009; Forrester 1968; Meadows 2008). This is accomplished by revealing feedback structures, which are loops in a system where a change in one part of a system creates effects that either reinforce (i.e., amplify) or balance (i.e., stabilize) that original change (Forrester 1968; Meadows 2008). Understanding feedback mechanisms can help identify leverage points, places in the system where change made will have a relatively large systemic impact (Meadows 2008).

Given their central role in system dynamics, it is important to clarify systems and feedback loops. Meadows (2008) defined a *system* as, “…an interconnected set of elements that is coherently organized in a way that achieves something” (p. 11). To be a system, something must have elements, interconnections, and a function (material systems) or purpose (human and combined systems; Meadows 2008). Elements are individual components of a system (e.g., students, classes, values, emotions), interconnections are relationships between elements (e.g., students connected to faculty, emotions connected to an observation), and a system's purpose comes from its behavior and action, not from what humans desire its purpose to be (e.g., classroom system may produce students focused on grades, not necessarily learning outcomes; Meadows 2008).

*Feedback* occurs when elements and their interactions compound one another, leading to patterns in system behavior (Meadows 2008). There are two types of feedback mechanisms: reinforcing loops and balancing loops. A *reinforcing loop* enhances the direction of change as more is added to the system, amplifying behavior (Meadows 2008). Reinforcing loops can be positive (e.g., the *more* I engage in my department, the *more* visible I am, the *more* I get asked to engage) or negative (e.g., the *less* time I spend grading, the *lower* the quality of feedback I give, the *less* students read my feedback, so I spend *less* time grading). Regardless, reinforcing loops drive more of the same within a system. A *balancing loop* seeks to stabilize system behavior (e.g., the *more* students we recruit, the *fewer* resources we have for our students, so we *reduce* our recruitment; Meadows 2008), sometimes beneficially and other times detrimentally. Neither of these loops has inherent goals but are instead indicators of system behavior.

In essence, system dynamics involves modeling systems to better understand what is happening within them. System dynamics provides a means of making mental models explicit, translating what is kept in the mind to a physical medium (Vennix 1996). These models often include input from different people to reflect a range of experiences and perspectives across a system. Although models can't predict the future with certainty, they can show how a system might respond under certain conditions (Ford 2009). Like a weather forecast, these projections can be off, but they still help us see patterns, test ideas, and think through complex problems more clearly.

## 3 Tools for Visualizing Systems

The adage “a picture is worth a thousand words” cannot be more relevant than when describing complex problems or systems. Just try to begin using words to describe problems like climate change or systems like pre‐K through 16 education and things start to get jumbled quickly. Due to their intertwined, vastly connected, and dynamic nature, complex situations are not easily described with linear methods. Employing visual strategies can help people make sense of the mess (Bell et al. 2016). Below, we describe rich pictures and CLDs, two tools we find helpful to make sense of complexity in our research and practice.

### 3.1 Rich Pictures

Originally created as a component of soft systems methodology (Checkland 1999), the rich pictures process encourages participants to make their thinking about a problem or system explicit (Bell et al. 2016). As such, rich pictures are visual representations of mental models. They can help capture nuance and demonstrate relationships between elements through imagery and metaphor, allowing participants to convey abstract or taboo ideas (Bell et al. 2016). Whether drawn by hand or created with the help of technology, rich pictures provide an accessible entry point for helping participants gain a more nuanced understanding of their problem and its context (Lewis 1992).

#### 3.1.1 Rich Picture Creation

The process for creating a rich picture can be as simple as an individual drawing their perspective of a situation on a napkin or as complex as a professionally facilitated, multi‐day workshop with stakeholders representing disparate parts of a complex system (Bell et al. 2016; Armson 2011). Regardless of the context, the essence of rich picture creation is the same. Provide people with something to draw on, something to draw with, space to consider their experiences and observations of a situation, and time to represent it.

There are no concrete “rules” for creating rich pictures; however, we do suggest three guidelines based on our experiences and those of other scholars and practitioners experienced in rich picture processes. First, focus on the *situation*, not the *problem* (Armson 2011; Bell et al. 2016; Gisby et al. 2023). As you will see from the following example, when we think about rich pictures as a tool to support problem‐solving, we may be tempted to focus our attention on the problem as we see it. However, approaching rich pictures as a way to think about the situation in which the problem is contextualized affords participants two main advantages. First, it allows participants to engage in problem *structuring* by helping participants unpack the issue and recognize other contributing factors (Bell et al. 2016), leading to a clearer understanding of which problem is the “right” problem to address (Armson 2011; Bell et al. 2016). Second, this wider perspective avoids narrowing solutions to one interpretation, instead opening space for more creative and effective interventions (Armson 2011).

**FIGURE 1:**
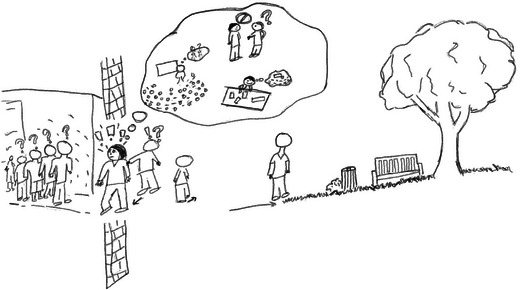
Rachel's rich picture.

To illustrate the value of the first guideline, we recall your attention to Rachel who is frustrated by a lack of student motivation and initiative in her community‐engaged class project. Rachel independently created a rich picture illustrating her perspective of the problem (See Figure 1). Rather than focusing on the problem of student motivation, Rachel expanded the focus of her picture to the entire community‐engaged project situation. In doing so, she included her perceptions of community members (on the right side of the drawing) as well as her own thoughts and emotions in the process. With this perspective, Rachel was able to articulate the pressures she feels from students, community members, and her other professional obligations while trying to facilitate the project. Had Rachel focused her drawing on student motivation, she may not have identified this feature of the problem.

A second suggested guideline for creating rich pictures is to avoid using text (Armson 2011; Bell et al. 2016). Text in the rich picture may reduce your ability to see patterns or relationships and may oversimplify the meaning to be communicated (Armson 2011). In Rachel's example, we see a thought bubble referencing things occupying her mind. Rachel could have written the word “teaching” to represent her interactions with students. Instead, she drew herself in action—teaching in a lecture‐style hall, separated from students, and thinking about an interaction she had with her supervisor. The richness of information conveyed in her drawing far surpasses what she could convey with a word and may help Rachel notice a pattern of separateness and having her mind elsewhere, conveyed in other parts of the drawing. Some educators/facilitators or participants may feel some text is necessary to convey their meaning. For example, it may be difficult for some participants to convey an emotion through drawing, so they may use an exclamation like, “Eek!” Other participants may choose to label a group of people in their drawing as “students” or “administrators” to improve clarity in interpretation.

A third suggested guideline for creating rich pictures is to lean into the use of symbols and visual metaphors (Armson 2011; Bell et al. 2016). People commonly use symbols and metaphors in daily communication; we give a “thumbs up” to indicate our agreement or say we are “swamped with meetings.” Each symbol or metaphor allows us to convey meaning in simplified ways. The same benefit is realized by including symbols and metaphors in rich pictures. In Rachel's rich picture, she draws herself in a brick wall between students and community members. This use of visual metaphor illustrates Rachel's feeling of being an impassable barrier between students and community members. Bell et al. (2016) suggested visual metaphors like the one illustrated by Rachel, allow participants to convey strong emotions or potentially contentious perspectives, bringing to light facets of the situation which may be difficult or undesirable to articulate explicitly.

It is important to remember how symbols and metaphors can carry multiple meanings and are interpreted by the creators and the viewers. A simple example is the check mark symbol. Some may interpret the symbol to mean “done” or “complete,” while others may interpret the symbol to mean “good.” Other symbols may be interpreted differently based on experience or culture. Therefore, when using rich pictures, understanding the intended meaning conveyed in the imagery is essential. Some meaning may be derived from context within the drawing—a water droplet near a cloud likely represents rain, while a water droplet near a sad face likely represents tears. However, not all symbols and metaphors are as easily deciphered through context. For this reason, rich pictures created in groups or by individuals other than the end user are often described to the larger group, educator, facilitator, and/or researcher.

#### 3.1.2 Facilitating the Rich Picture Creation Process

As with the creation of rich pictures, there are no concrete “rules” for facilitating a rich pictures process. Rather, rich picture experts assert that the facilitation process should be flexible, allowing each facilitator to find a process that works for them and the goals of the session (Bell et al. 2016). The example we share in this section emphasizes group creation and draws primarily from our own experiences facilitating rich pictures sessions, while incorporating suggestions from Bell et al. (2016) and Armson (2011).

Rachel is interested in hearing from her community partners about how the community‐engaged class project works for them, so she invites each partner to share their perspectives through a rich picture. Knowing the environment, she chooses will influence how her community partners engage (Bell et al. 2016), Rachel reserves a room in a neutral space. She also prepares drawing materials for collaborative groups of four to six participants (Bell et al. 2016).

Rachel knows one of the most difficult hurdles for rich picture creation is convincing participants that drawing pictures is (1) valuable, and (2) something they can do (Bell et al. 2016). She begins the session with a quick overview of the value of pictures in making shared meaning, then encourages participants to “warm‐up” by trying out some doodles in a corner of their flipchart paper. Once it seems like participants are warming up to the process, Rachel gives instructions for creating the collaborative rich pictures. Although some scholars/practitioners offer specific guidance for the process of creating rich pictures (Armson 2011) or specific icons to be used in their creation (Berg and Pooley 2013), Rachel chooses to take a more hands‐off approach to facilitation (Bell et al. 2016). She provides her participants with a prompt, “Draw a picture about your experience as a community partner in the LEAD 326 student leadership projects.” Then, Rachel suggests groups take time to think, discuss their experiences, and collaborate on their drawing when they are ready.

As each group begins to discuss and draw, Rachel patiently responds to questions, encouraging participants to take ownership of their drawing. She emphasizes the importance of the process as a way for the group to make their own interpretation of the experience (Bell et al. 2016). Soon, Rachel begins to fade into the background as participants gain more autonomy. However, she might choose to join the group of three to add another perspective. She might notice a member of one group becoming authoritative and decide to intervene, taking a more active facilitation role to ensure all participants have space to share. Alternatively, Rachel could have decided from the outset she would lead the whole group in creating the rich picture, creating the drawing while community partners shared their experiences. To reiterate, there is no “one right way” to create a rich picture. The facilitator has flexibility to decide the best way to achieve the group goals given their constraints.

**FIGURE 2:**
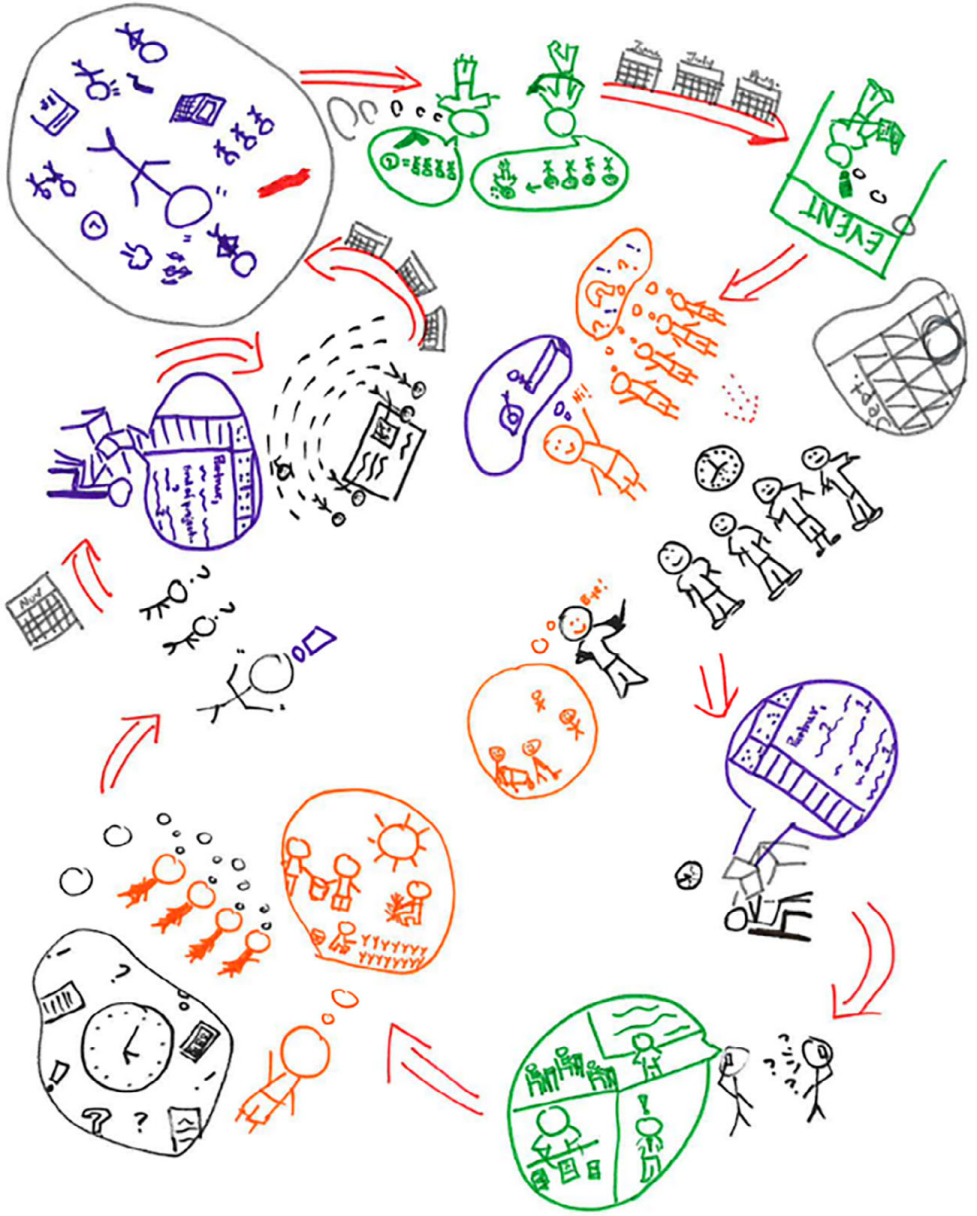
Community partners’ rich picture. This drawing is a representation of a rich picture which may be co‐created by stakeholders.

After about 40 min of collaborative drawing time, Rachel asks participants to display their rich pictures in a highly visible space in the room and share their creations with the other participants. See Figure 2 for an example created by one group. Although group members are sharing, Rachel invites other group members to clarify or expand upon specific symbols or visual metaphors. For example, Rachel sees multiple clocks in the drawing and asks participants to share the significance. This process allows all community partners to learn about each group's perspective and supports Rachel in interpreting the rich pictures when she revisits them after the session.

#### 3.1.3 How to Use Rich Pictures

The creation of a rich picture provides two primary outcomes for problem solving: (1) the meaning‐making and group learning that occur and (2) the picture itself (Bell et al. 2016). Through the process of creating her individual rich picture, Rachel was able to organize her thoughts about the problem, now seeing as a holistic picture what was a cluster of disjointed observations. Further, by engaging her community partners, Rachel expanded her understanding of the problem and connected the community members with one another, encouraging sharing about their collective experience.

The group learning and meaning‐making which emerged from the rich picture session helped each participant expand their mental model of community‐engaged course projects and may inspire a change in action when asked to engage again. In addition to the process of engaging, Rachel now has multiple artifacts which represent different perspectives of the same problem situation. After re‐examining and comparing the pictures, Rachel can use the content and patterns represented to identify problems which are most useful to address.

#### 3.1.4 Rich Picture Conclusions

In our hypothetical case study, Rachel experienced a persistent problem of lack of student motivation to engage in the community‐based course project. By making her own rich picture, Rachel identified a pattern of thinking of other professional responsibilities while working with students and stakeholders, and articulated feeling like a barrier between students and community members. When Rachel facilitated a group rich pictures session with community partners, the participants also expressed a heavy reliance on Rachel to answer student questions. Noticing this pattern across perspectives gave Rachel an idea for a joint information session and more open communication channels between students and community partners.

Broadly, rich pictures are a valuable tool for individuals or groups to make sense of a problem and the context in which it is situated. By creating a visual representation of how they perceive the problem, participants can sort through the structures, resources, emotions, and other facets of the problem to gain a more nuanced understanding of how it is situated in the specific context. Further, when the creators of rich pictures make their perspective of the problem visible to other stakeholders, the shared meaning provides the group with common ground from which to begin addressing the problem in a way that accomplishes the overall goal.

### 3.2 CLDs

CLDs are primarily heuristic tools (Voinov et al. 2018) which allow the creators to represent a specific part of a problem or system as a series of interconnected elements. Importantly, CLDs have clearly established boundaries for each system they are meant to depict; there will be aspects which may be important to a system that lie outside of the one being depicted. For example, if you were modeling student leadership opportunities within a high school, your model would focus on what is offered at the high school. However, it may be impacted by a local technical education center that students can access. A CLD will often precede more technical models of the same diagram, though CLDs may be standalone representations of systems as well (Lane 2008, as cited in Voinov et al. 2018).

CLDs give modelers insight into primary structures and suspected causal relationships within systems. CLDs are often built from participant experiences, using experiential knowledge to inform how a CLD will manifest. Furthermore, CLDs can be especially useful in hard‐to‐quantify systems, like the dynamics of student motivation or classroom engagement. Although CLDs are useful tools, they are not as powerful as more complex models, often missing underlying aspects simulated models may be able to capture (i.e., accumulation of a resource over time [Richardson 1986, 1997; Vennix 1996] and/or strength of feedback loops [Turner and Goodman 2023]).

CLDs are composed of elements, interconnections, polarity symbols, and feedback loops. Each of these concepts has a symbolic representation, as outlined in Table 1 (Marzolino and McKim 2024).

**TABLE 1:** Symbols used in causal loop diagrams.

Symbol	Meaning
+	Direct relationship between two variables (e.g., as one increases, the other increases).
—	Inverse relationship between two variables (e.g., as one increases, the other decreases).
→	Relationship between two variables.
R	Reinforcing feedback loop.
B	Balancing feedback loop.

*Note*: Modified from Marzolino and McKim (2024).

#### 3.2.1 CLD Creation

When preparing to create a CLD, methods can and should be tailored toward the context most relevant to the system being modeled. For example, if a community organization is trying to identify how to best eliminate barriers to food access, it would be good to have a variety of individuals within the community, organization, and tangential roles as participants in creating a CLD. In this context, it may be advantageous to prepare a workshop to bring people together. If one is curious about their tendency to misplace their favorite pen; however, they may only need to consult themselves while creating their CLD.

Materials needed for creating a CLD are simple: something to write on and a writing utensil, or somewhere to draw electronically. Datasets, personal devices, and more (e.g., the ability to project if making a shared digital model) may also be useful aids for individuals in thinking through elements and interconnections in CLDs; these additional aids may be relevant to some groups more than others. Additionally, there are software programs which allow for digital modeling (i.e., Stella, Vensim, etc.), but these advanced tools may not be necessary for the average person and their needs.

Starting a CLD will vary, depending on the context of the application. We recommend beginning by clearly defining the system of interest and setting some boundaries for the system at hand. As an example, Rachel is interested in the dynamics of student motivation. What influences student motivation to engage in community‐based activities, and how can she encourage more engagement? She would be interested in her own students’ experiences and feelings, allowing for a clear boundary on this system.

Once system definition and boundaries are established, the CLD can be created. It is considered good practice to denote where connections come from as they arise (Jalali and Beaulieu 2023). In Rachel's case, she could attribute different connections to students who posit those interconnections may exist. If modeling in an informal context, this may not be a necessary step; however, if the model is to be shared with others, these notations help to build credibility and trust (Jalali and Beaulieu 2023).

As a model is being visualized, there is no right or wrong way to go about expressing connections. New ideas often arise from cognitive dissonance throughout the process, which will result in model revisions. Allowing time for reflection will help participants check the CLD for credibility. This is often an iterative process, with the model being consistently refined, during the session, until it satisfies modeling participants.

Member‐checking for validity (Fine and Torre 2019; Lincoln et al. 2018) after model creation is recommended to see if the model is congruent with perspectives of other members of the field, or if it could use some additional modifications. In Rachel's example, one class session could be used to create the original model, allowing time for reflection afterward. Then, when class reconvenes, she could ask students to share anything more they thought to add or modify.

#### 3.2.2 Facilitating the CLD Creation Process

In the previous section, we describe general best practices for creating CLDs. In this section, we demonstrate CLD creation through an example, emphasizing how an educator or facilitator might support the process. In the example, Rachel is ready to convene with her students and depict a system pertaining to student motivation. First, she sets the stage for students, detailing the importance of understanding barriers to motivation. Next, she clearly defines the problem and sets system boundaries—her students, her class experiences with the community‐engaged project. Now, she's ready with a whiteboard and marker, hoping to capture student experiences. The first variable to mark the page is the core variable, student motivation, which she has written as “student willingness to engage”.

Though Rachel hopes her students will be quick to share, she notices blank stares and hesitancy. Knowing complex problems are not always linear, and some elements and interconnections may be challenging to access, Rachel decides to prime her students for the experience. To get her students talking and ideas flowing, Rachel breaks students into small groups for discussions focused on student experiences and connections to the core variable. She knows this will elicit students’ lived knowledge and help them tease it apart, making their mental models explicit.

**FIGURE 3:**
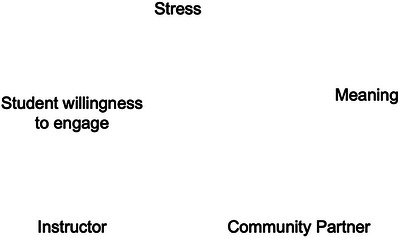
Causal loop diagram model creation example: Elements. Elements coming together during the first phase of CLD creation. Five is an arbitrary number of starting variables chosen to illustrate this process.

Once students are primed, they are quick to suggest different elements of the system, the individual pieces which impact their motivation. Rachel documents these elements on the board, beginning with instructor, community partner, stress, and meaning; see Figure 3.

**FIGURE 4:**
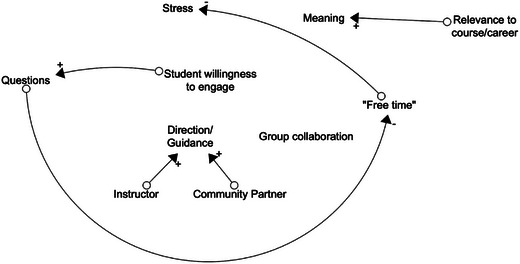
Causal loop diagram model creation example: Connections and polarity.

Students discuss and begin to identify more elements of the system, as well as some connections they're beginning to see. As students identify connections, Rachel asks them what the relationship between the variables is: As A increases, what does B do? This allows her to discern the polarity of connections. For direct relationships where A increases and B does as well, the arrow is marked with a plus. For inverse relationships where A increases and B decreases, the arrow is marked with a minus. See Figure 4 for examples.

**FIGURE 5:**
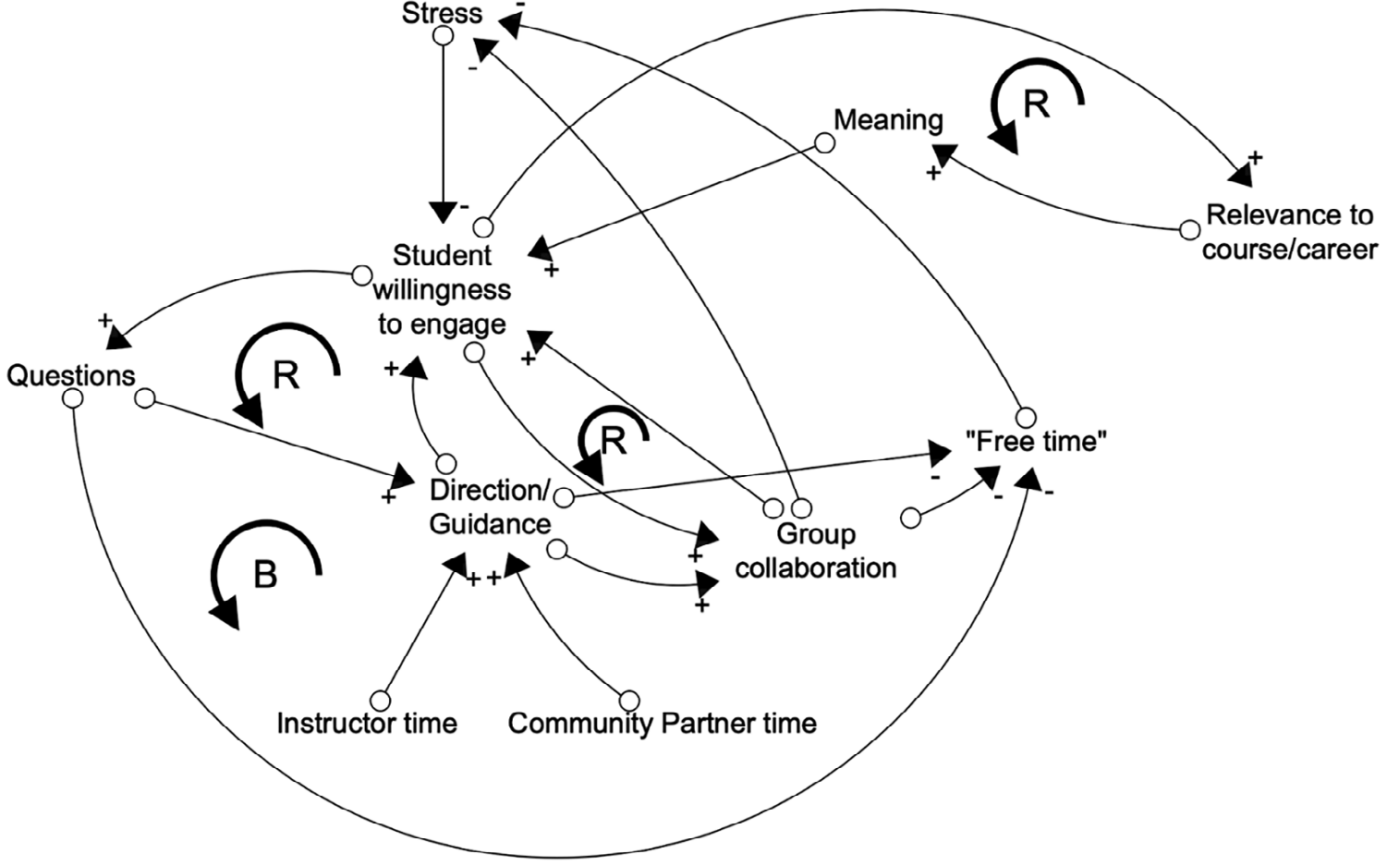
Causal loop diagram model creation example: Feedback loops.

As more interconnections are spotted, and loops begin to form, Rachel labels them appropriately: R for reinforcing, B for balancing. Additionally, the group becomes confused with the labels of “Instructor” and “Community Partner”. What they are talking about is not the number of instructors or community partners; it is the time they are spending on providing direction/guidance. Additionally, group collaboration needs to be defined. Are they working well together as a group? The students decide they are, so group collaboration is assumed to be when each group member is pulling their own weight. Rachel takes opportunities throughout the session to clarify these variables. Before long, student chatter dies down and Figure 5 results.

#### 3.2.3 How to Use CLDs

Once class is dismissed, Rachel sits with this CLD to try to make sense of it. The loops captured during class suggest there is a reinforcing loop between student willingness to engage, relevance to course/career, and meaning. This loop demonstrates the more relevant a partnership is, the more meaning it has to students; when that happens, students are more willing to engage. There is also a reinforcing loop between student willingness to engage and group collaboration; as groups are better collaborators, students are more willing to put in the work to get things done. Next, there is a reinforcing loop between student willingness to engage, questions, and direction/guidance; as students seek to engage, they ask questions, which requires more guidance from the instructor and community partner. Finally, there is a balancing loop they've found between student willingness to engage, questions, “free time”, stress, and student willingness to engage. This loop suggests that as students are more willing to engage, they ask more questions, which eats away at their “free time”, leaving them more stressed, and thus less willing to engage. This balancing loop intuitively feels that it could be the problem in Rachel's classroom.

As she scrutinizes this model, Rachel realizes there are a few loops which are missing or confusing. She feels there may be variables that were missed when they were co‐creating this model. She brings this model back to class and talks through it with students, hoping to improve this visualization. She also explains the model to students, and they argue it feels true to life. At this point, Rachel begins to wonder what action she can take to intervene within this system.

#### 3.2.4 CLD Conclusions

In Rachel's example, she has been able to identify where she would like to intervene, choosing to dedicate more of her time to coordination and streamlining projects to minimize student questions. By taking this action, she hopes students will have more free time and less stress, allowing them more willingness to engage. In Rachel's case, the CLD created alongside students has given her some hope for the future and a better understanding of her students and their motivation.

Broadly, CLDs are a tool that are immensely helpful for visualizing and examining dynamics of systems. By doing so, practitioners may identify leverage points—areas in the system where a small change can make a big difference—and intervene to alter system behavior. These models have various applications and are incredibly versatile, transcending disciplinary boundaries and capturing the complex as well as the mundane. However, while these tools have their uses, they may not be as powerful as simulated models. Context and application are important considerations for deciding which tools to use for visualizing systems.

## 4 Practical Considerations

Rich pictures and CLDs are two valuable tools for visualizing complex systems, each with its own unique strengths. A comparison of rich pictures and CLDs is provided in Table 2. Further, rich pictures can be a valuable starting point for creating a system map (Armson 2011), like a CLD. For example, a participant may identify a theme in a rich picture they want to explore further, so they create a CLD to represent the dynamics at play. Some elements of the CLD may be derived from the rich picture while others may emerge independently. Although the two tools work well in tandem, rich pictures are not the only entry point into the creation of CLDs, as described in the previous sections.

**TABLE 2:** Comparison of rich pictures and causal loop diagrams.

	Rich pictures	Causal loop diagrams
Type	Imaginative	Heuristic
Purpose	Identify elements of and connections related to a problem of interest	Identify patterns and make sense of interconnection between variables
Origins	Soft systems methodology (Checkland 1999)	System dynamics modeling (Forrester 1968; Meadows 2008)
Scope	Used to explore the situation surrounding a problem or issue	Used to explore a specific problem or issue within specified boundaries

### 4.1 Common Questions in Visualizing Complex Systems

Complex systems are messy, continuously evolving, and often include different groups or individuals with different perspectives or goals. These characteristics make systems work challenging for many, especially scholars and practitioners who are new to systems thinking. As such, we share insights into three common questions about visualizing complex systems.

#### 4.1.1 How Do We Know When We're Done?

It can be difficult to know when a rich picture or CLD is finished. The creator(s) of either might wonder if an element is too trivial or too broad to include, they might step away only to think of another important element to add, or they might speak with a colleague or student who inspires another important element of the system. When this occurs, it is important to remember each model of the system is simply a representation. Iterations of a rich picture or CLD are encouraged to support the quality and credibility of the picture or diagram (Armson 2011; Bell et al. 2016; Gisby et al. 2023; Meadows 2008). A quality and credible visual representation of a system is one that is “accepted as representative of the situation, realistic and useful” (Walker et al. 2014, p. 356).

#### 4.1.2 Who Needs to Be Involved?

Although rich pictures or CLDs can be created by individuals, for some problems, their power is increased when created collaboratively. This makes each an especially useful tool for use with participatory and action research methodologies. Complex problems and systems typically involve many stakeholders, each with a unique perspective of the context. When engaging stakeholders at any point in the research process, misunderstandings and conflicting visions of the problem or solutions can quickly deteriorate progress. Therefore, it is helpful for stakeholders to have a shared understanding of the problem from the lens of the whole group. The co‐creation of rich pictures and CLDs from the outset can help participants create this shared understanding, reducing hurdles in future action.

#### 4.1.3 How Do We Handle Disagreement and Conflict?

Disagreement and conflict in any collaborative or participatory project are not signs of failure. Instead, they demonstrate participants care about the problem and are comfortable contributing their unique perspectives to the discussion. Lack of disagreement may indicate groupthink or other consensus‐seeking behaviors in which individuals act on the will of the majority to prevent tension or disruption (Bell et al. 2016).

As facilitators of a rich pictures or CLD session, we may remember our role is to create a welcoming environment which encourages equitable interaction and engagement among participants. Therefore, as disagreements and conflict arise, facilitators can help participants explore their own and others’ perspectives in a way that encourages participants to find a mutual purpose (Grenny et al. 2022). In doing so, facilitators should also be aware of power dynamics among group members, recognizing some participants may use their power to covertly exclude others or control the narrative of the group (Gaventa and Cornwall 2015). It is also helpful to establish expectations for engagement at the beginning of any project and/or work session (Arieli et al. 2009) and co‐address any conflict or misalignment of goals or values early in the process (Avila et al. 2018).

## 5 Conclusion

Throughout this paper, we explored a fictional story about Rachel, a leadership educator who struggled with student motivation in her community‐engaged course project. Rachel's story helps us demonstrate the value and utility of rich pictures and CLDs, two tools for visualizing systems. The character and story we used in this paper were fictional, but the value of the tools are not. The landscape of leadership education is rife with complex problems and situations. Whether you are a student struggling with time management, faculty or staff member seeking sustainable funding sources, or an administrative leader striving to promote harmony among your unit, system visualization tools like rich pictures and CLDs help make sense of the mess. With patterns, connections, and feedback loops illuminated, you can see small changes that can help address big and complex problems.
